# Effects of PEG-Induced Water Deficit in *Solanum nigrum* on Zn and Ni Uptake and Translocation in Split Root Systems

**DOI:** 10.3390/plants4020284

**Published:** 2015-06-05

**Authors:** Urs Feller, Iwona Anders, Shuhe Wei

**Affiliations:** 1 Institute of Plant Sciences and Oeschger Centre for Climate Change Research, University of Bern, Altenbergrain 21, CH-3013 Bern, Switzerland; E-Mail: iwona.anders@ips.unibe.ch; 2 Key Laboratory of Pollution Ecology and Environmental Engineering, Institute of Applied Ecology, Chinese Academy of Sciences, Shenyang 110016, China; E-Mail: shuhewei@iae.ac.cn

**Keywords:** drought, transport, xylem, phloem, split root system, cations, heavy metals, radionuclides, *Solanum nigrum* L.

## Abstract

Drought strongly influences root activities in crop plants and weeds. This paper is focused on the performance of the heavy metal accumulator *Solanum nigrum*, a plant which might be helpful for phytoremediation. The water potential in a split root system was decreased by the addition of polyethylene glycol (PEG 6000). Rubidium, strontium and radionuclides of heavy metals were used as markers to investigate the uptake into roots, the release to the shoot via the xylem, and finally the basipetal transport via the phloem to unlabeled roots. The uptake into the roots (total contents in the plant) was for most makers more severely decreased than the transport to the shoot or the export from the shoot to the unlabeled roots via the phloem. Regardless of the water potential in the labeling solution, ^63^Ni and ^65^Zn were selectively redistributed within the plant. From autoradiographs, it became evident that ^65^Zn accumulated in root tips, in the apical shoot meristem and in axillary buds, while ^63^Ni accumulated in young expanded leaves and roots but not in the meristems. Since both radionuclides are mobile in the phloem and are, therefore, well redistributed within the plant, the unequal transfer to shoot and root apical meristems is most likely caused by differences in the cell-to-cell transport in differentiation zones without functional phloem (immature sieve tubes).

## 1. Introduction

During extended drought periods, species composition and productivity of grasslands are affected [1,2,3,4,5,6]. Plants are often exposed to strong soil water potential gradients [7,8]. Therefore, roots from the same plant may be in soil regions differing in their water availability. Experiments with split root systems might be helpful to elucidate interactions between differently stressed roots and the shoot. Markers applied to a part of the root system can be used to elucidate the uptake into the roots, the root-to-shoot transfer, and finally the basipetal transport via the phloem. Since the two parts of the root system can be subjected to different water potentials, such experiments may allow a deeper insight into the uptake into the roots, the transfer of the labels in the transpiration stream via the xylem to the shoot and the symplastic transport from the shoot to the other part of the root system via the phloem.

Organic compounds are not first choice as markers for such transport experiments, since they may be metabolized, insolubilized or converted to volatile compounds and released in gaseous form. Suitable markers should be stable, not metabolized, not phytotoxic and easily detectable (preferentially various labels in the same plants). Rubidium and strontium are useful non-radioactive labels, since they are not metabolized. Rubidium (similar to the macronutrient potassium) is highly mobile in xylem and phloem and is therefore well redistributed within the plant [9,10,11]. Strontium (similar to the macronutrient calcium) is loaded into the root xylem, reaches the shoot via the transpiration stream and accumulates in transpiring organs, since this element is characterized by a very poor phloem mobility [11,12,13]. Radionuclides of heavy metals were successfully used in the past for translocation experiments in wheat [11,14,15], white lupin [16] and *Solanum nigrum* [17]. The release of several heavy metals (Cd, Co, Mn, Ni and Zn) from the roots to the shoot, the further redistribution within the shoot and the shoot-to-root transport in the phloem differed considerably between *Triticum aestivum* [11,14,15], *Lupinus albus* [16] and *Solanum nigrum* [17].

Various plant species or even varieties of the same species may be affected differently by drought [1,3,4,5,6,7,8,18,19]. As a consequence, the species composition in pastures may be altered during, and also for some time after, a summer drought [3,4,5,6,7,8]. Impacts of extended drought periods on edible crop plants [19,20,21,22,23,24,25], fodder plants [1,6,7,8,26,27,28] and weeds [3,29] were reported previously, but heavy metal hyperaccumulators were so far not the focus. *Solanum nigrum* was identified as a hyperaccumulator of Cd and other heavy metals [30]. This species might be helpful for the bioremediation of contaminated soils, although it is less efficient in cadmium accumulation than *Sedum alfredii* or *Thlaspi caerulescens* [30,31,32,33,34]. *Solanum nigrum* is known for an efficient root-to-shoot transport of heavy metals making it suitable for cleaning contaminated soils [17,30]. Therefore, the response of this species to drought stress is of practical relevance. Furthermore, this species with special heavy metal transport properties may be an interesting model system for basic physiological studies referring to source/sink interactions under drought.

The aim of the investigations reported here was to identify in a split root system with *Solanum nigrum* the performance of the whole plant when only parts of the root system suffer from limited water availability considering (a) the uptake of cations into the roots, (b) the transport from the roots to the shoot with the transpiration stream in the xylem, and (c) further redistribution processes via the phloem within the shoot and between the shoot and the roots.

## 2. Results and Discussion

**Figure 1:**
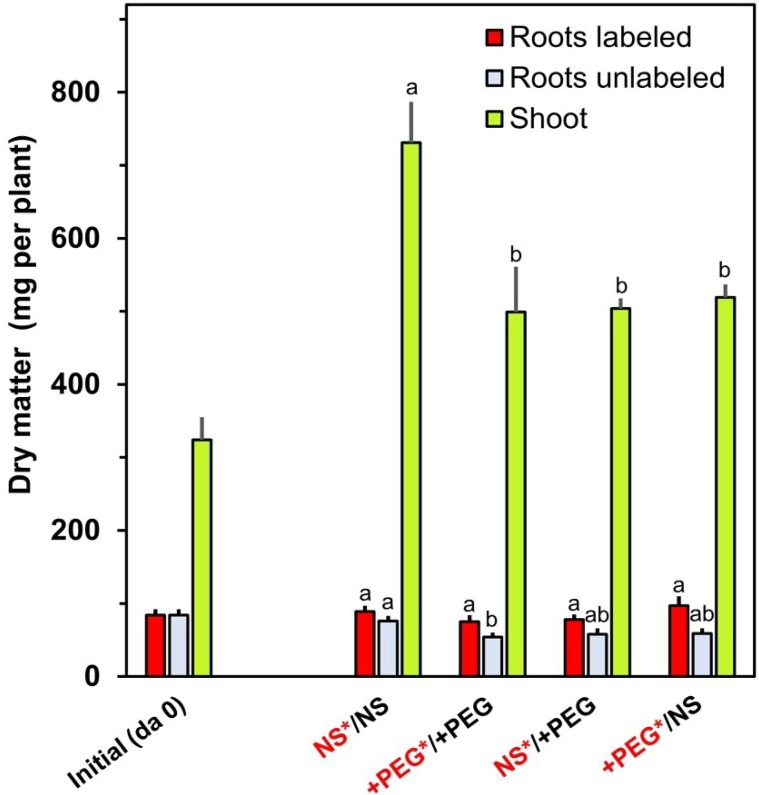
Dry matter in labeled roots, unlabeled roots and in the shoot before and after the exposure to the various treatments. Means+SE of six replicates are shown. Columns for a given plant part (roots labeled, roots unlabeled or shoot) with the same letter (a, b) are not significantly different at the *p* < 0.05 level. NS: nutrient solution. +PEG: nutrient solution with polyethylene glycol 6000. Red fonts: solution with labels.

The root biomass remained quite constant during the labeling period of 96 h, while the shoot biomass increased considerably (Figure 1). The shoot biomass at the end of the experiment was significantly higher in unstressed plants than in plants with one or both parts of the root system exposed to a lowered water potential. From these results, it became evident that the stress applied to the roots affected biomass production, although plants were still growing in all treatments.

**Figure 2:**
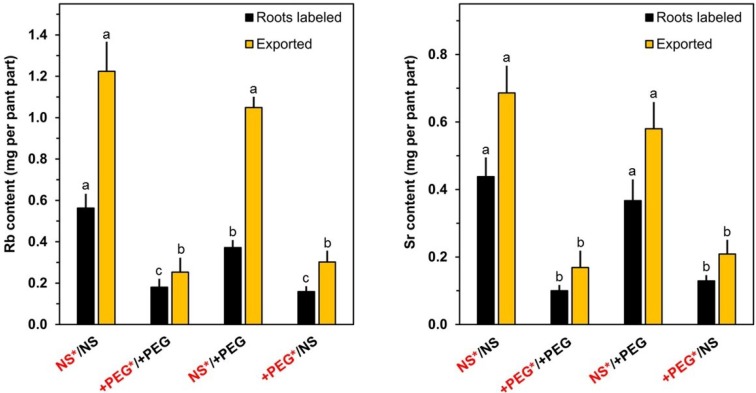
Contents of Rb and Sr in labeled roots and in the other plant parts (exported) after the incubation period of four days. Rb and Sr were added to the nutrient medium at the beginning of the PEG treatment (Day 0). Means+SE of six replicates are shown. Columns for a given plant part (roots labeled or exported) with the same letter (a, b) are not significantly different at the *p* < 0.05 level. NS: nutrient solution. +PEG: nutrient solution with polyethylene glycol 6000. Red fonts: solution with labels.

Rubidium (similar to potassium) and strontium (similar to calcium) were present in the nutrient medium in excess from the beginning of the stress period (Figure 2). The uptake into the plants via the roots is the first physiological step in the acquisition of these elements. The translocation from the labeled roots to the shoot via the xylem is another important aspect (Figure 2). The two elements were more rapidly taken up by roots in standard nutrient medium than by those in PEG-containing medium (sum of black and orange columns). The total export of rubidium from labeled roots to the shoot via the transpiration stream was considerably higher in the absence of PEG leading to a high shoot/root ratio for this element. The shoot/root ratios for strontium contents were in PEG-containing medium similar to those measured for rubidium, but a large portion of the strontium taken up was retained in the labeled roots incubated in PEG-free medium. From these results, it became evident that the effects of a lowered water potential on uptake and release from the labeled roots to the shoot must be addressed in an element-specific manner.

**Figure 3:**
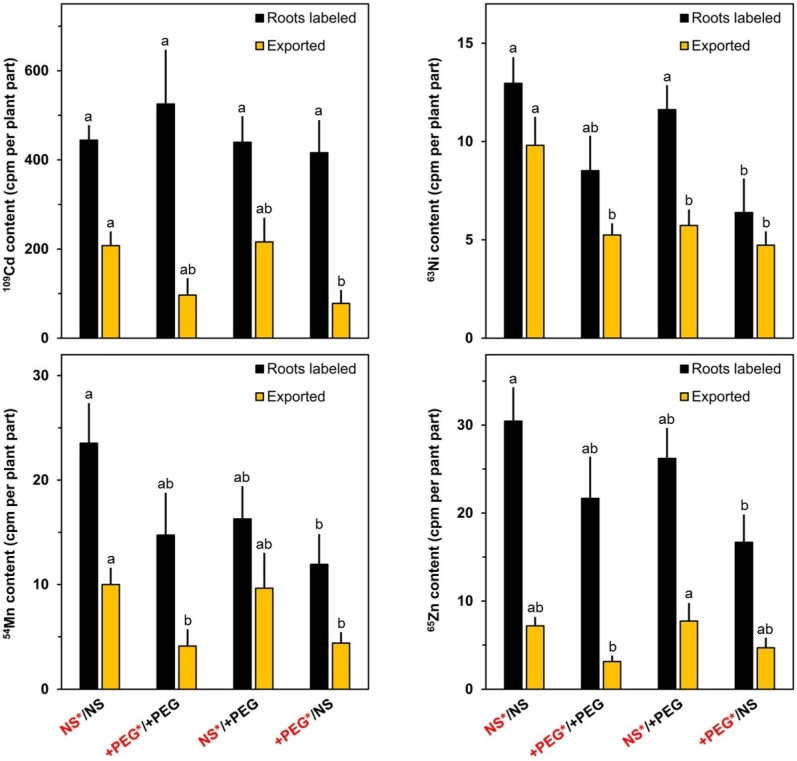
Contents of ^109^Cd, ^63^Ni, ^54^Mn and ^65^Zn in labeled roots and in the other plant parts (exported) after the incubation period of four days. ^109^Cd was added at Day 1 (24 h after start of treatment), ^63^Ni and ^54^Mn were added at Day 2 (48 h after start of treatment) and ^65^Zn was added at Day 3 (72 h after start of treatment). Means+SE of six replicates are shown. Columns for a given plant part (roots labeled or exported) with the same letter (a, b) are not significantly different at the *p* < 0.05 level. NS: nutrient solution. +PEG: nutrient solution with polyethylene glycol 6000. Red fonts: solution with labels.

The heavy metals were added sequentially to the nutrient medium of one half of the split root system to allow a better resolution of the overall effects of artificial drought throughout the stress period (Figure 3). A strong retention in the labeled part of the roots system was observed for ^109^Cd (added to the nutrient medium one day after the onset of the stress period). No significant differences between the various treatments were observed for the content of this radionuclide in the labeled roots. The export from the labeled roots was decreased when the water potential in the medium of the labeled roots was decreased, indicating that the root-to-shoot transfer was negatively influenced by artificial drought (similar to rubidium and strontium). The uptake of ^63^Ni from the medium into the roots was decreased in drought-stressed plants. A high percentage of ^63^Ni was released from the labeled roots to other plant parts under all experimental conditions. The largest quantities of ^63^Ni were exported from the roots of unstressed plants. ^54^Mn (added simultaneously with ^63^Ni to the medium two days after the beginning of the stress period) was less efficiently released from the roots to other plant parts than ^63^Ni, but the overall response to the lowered water potential was similar for both radionuclides. ^65^Zn was added to the nutrient medium three days after the onset of the artificial drought (one day before collecting and analyzing the plants). Although only a small quantity of this radionuclide was released from the roots to the shoot during this short period of one day, the overall response was very similar to that observed for ^109^Cd indicating that the release from the labeled roots was affected by drought in a rather unspecific manner for the radionuclides tested.

**Figure 4:**
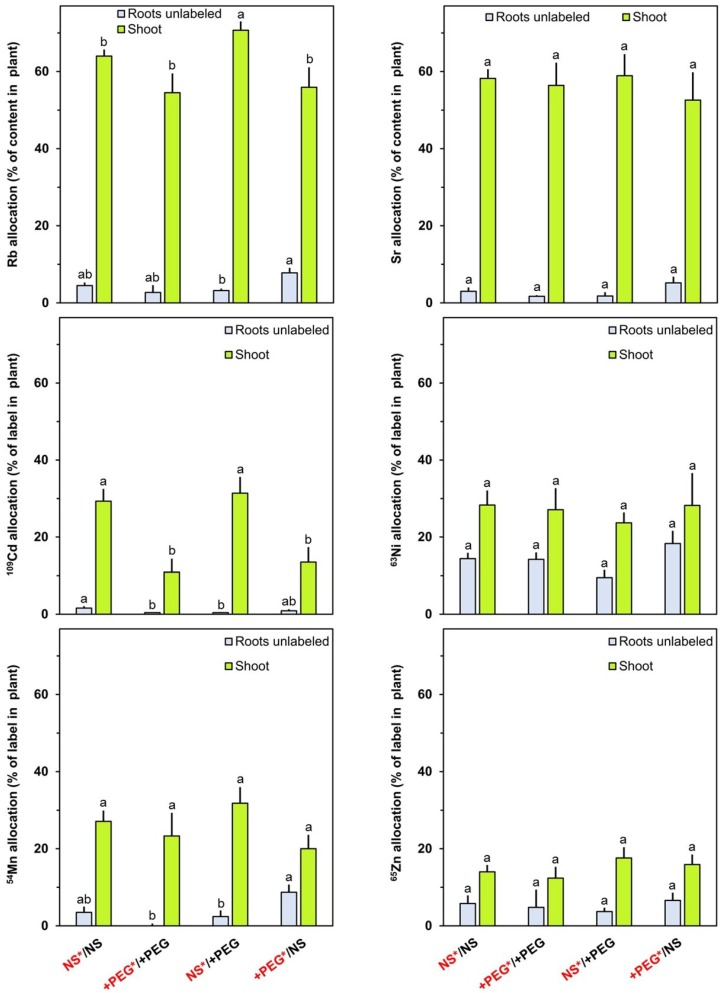
Allocation of Rb, Sr, ^109^Cd, ^63^Ni, ^54^Mn and ^65^Zn to the shoot and to unlabeled roots after the incubation period of four days in percent of the total content per plant. Rb and Sr were present from the beginning of the treatment (Day 0), ^109^Cd was added at Day 1 (24 h after start of treatment), ^63^Ni and ^54^Mn were added at Day 2 (48 h after start of treatment) and ^65^Zn was added at Day 3 (72 h after start of treatment). Means+SE of six replicates are shown. Columns for a given plant part (roots labeled or exported) with the same letter (a, b) are not significantly different at the *p* < 0.05 level. NS: nutrient solution. +PEG: nutrient solution with polyethylene glycol 6000. Red fonts: solution with labels.

The transfer from the roots to the shoot is based on the transpiration stream in the xylem and phloem mobility is not a crucial aspect in this context. However, the basipetal transport from the shoot to the unlabeled parts of the root system depends on phloem mobility. The percentage of the label in the plant transferred to the unlabeled parts of the roots system was highest for ^63^Ni followed by ^65^Zn which was added only one day before collecting the plants (Figure 4). For the other labels (Rb, Sr, ^109^Cd and ^54^Mn), the relative contents in the unlabeled roots were far smaller in the unlabeled roots than in the shoot indicating a poor basipetal transport.

**Figure 5:**
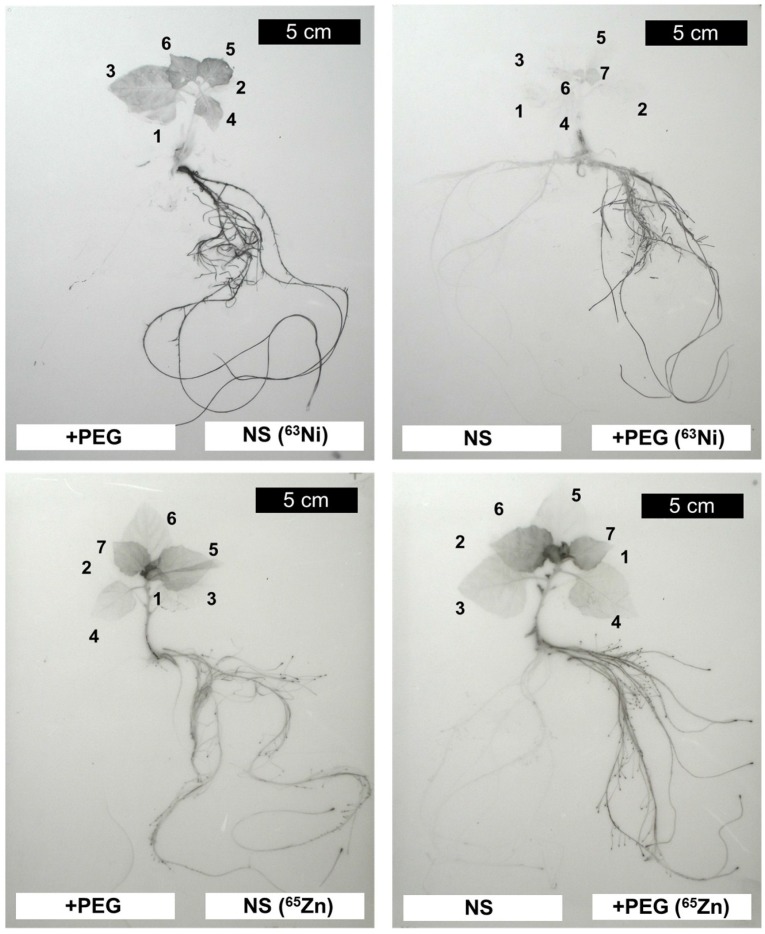
Autoradiographs of plants labeled for four days with ^63^Ni or with ^65^Zn in a split root system for two combinations of nutrient media. Cotyledons were no longer present at the beginning of the labeling phase. Leaves are numbered from the oldest (1) to the youngest (7). Only very weak signals were detected in the unlabeled roots and in the oldest two leaves (1, 2). NS: nutrient solution. +PEG: nutrient solution with polyethylene glycol 6000.

**Figure 6:**
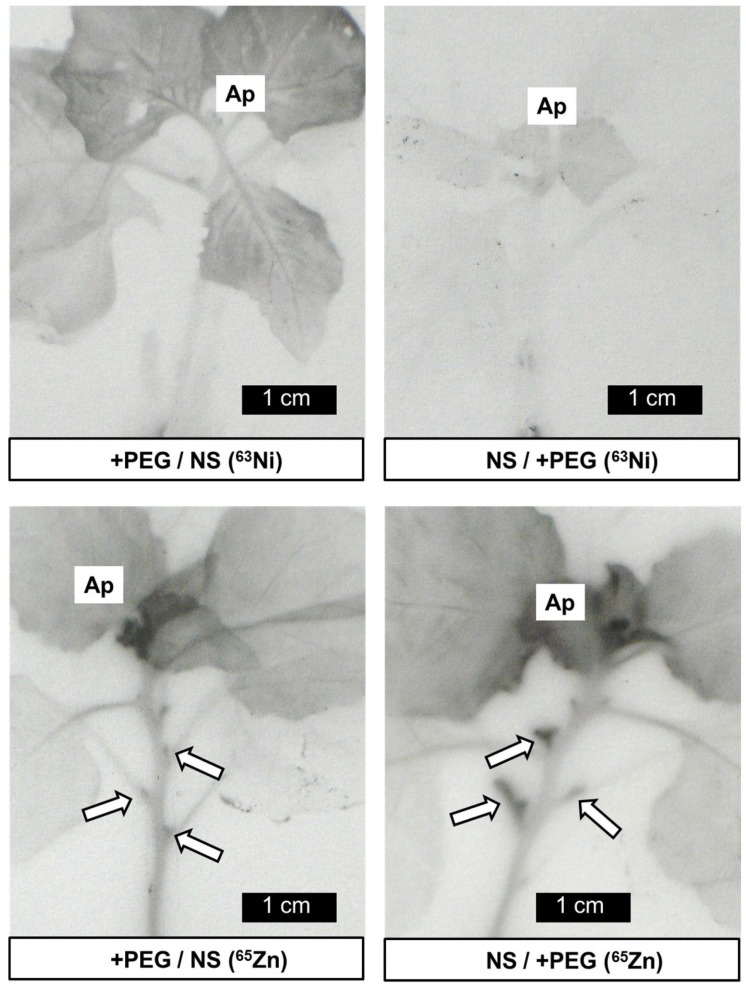
Autoradiographs of plants labeled for four days with ^63^Ni or with ^65^Zn in a split root system for two combinations of nutrient media (details from Figure 5). The apex (Ap) and axillary buds (arrows, for ^65^Zn only) are indicated.

**Figure 7:**
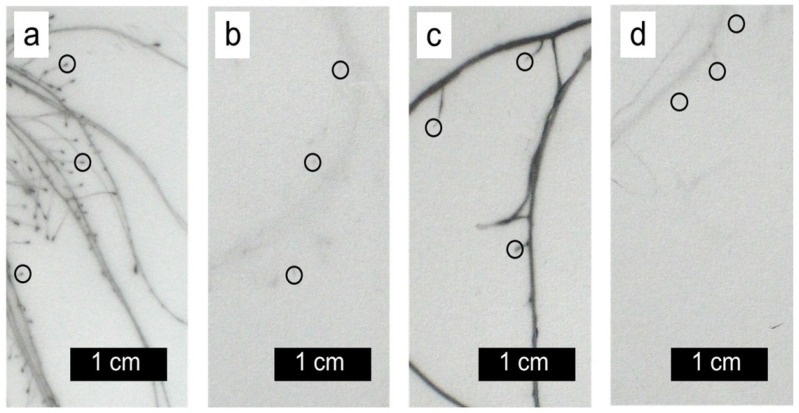
Autoradiographs (details from Figure 5) of labeled (**a**,**c**) and of unlabeled (**b**,**d**) roots in a split root system incubated for 4 days with ^65^Zn in (**a**,**b**) or with ^63^Ni (**c**,**d**). The root medium was nutrient solution with polyethylene glycol (a) or nutrient solution without polyethylene glycol (**b–d**). Some root tips are encircled. Only very weak signals were detected in the unlabeled roots.

The measurements mentioned above cannot answer the question how the various labels were distributed within the roots and within the shoot. Autoradiographs with ^63^Ni and ^65^Zn allowed a visualisation of the distribution patterns (Figure 5). Both radionuclides were detected in the labeled part of the root system and in younger leaves, while the unlabeled roots and the oldest leaves contained only traces. These findings are consistent with quantitative data mentioned above (Figure 2, Figure 3 and Figure 4). However, the relative labeling of the roots tips, the youngest and still very small leaves and the axillary buds differed for these two radionuclides. While ^65^Zn was strongly accumulated in meristems, this was not observed for ^63^Ni. To allow a better comparison, enlargements of shoot and root regions are shown in Figure 6 and Figure 7.

^63^Ni was mainly located in the young expanded and expanding leaves but not in the youngest leaves and in the meristems (Figure 6). In contrast, ^65^Zn was concentrated in youngest leaves and in axillary buds (Figure 6). The different distribution patterns for the two radionuclides were observed regardless of the presence of PEG in the labeling solution. A strong accumulation of ^65^Zn (but not of ^63^Ni) was observed in the tips of the labeled roots (Figure 7). The accumulation of ^65^Zn in the root tips was also observed in newly formed and in unlabeled roots, indicating that it was not just a consequence of a high uptake rate in the tip. ^63^Ni and ^65^Zn were reported to be highly phloem-mobile in various plants [14,15,16,17]. A possible explanation for the different distribution of ^63^Ni and ^65^Zn in roots and shoots could be based on differences in the cell-to-cell transport in plant parts not yet containing a functional phloem (meristematic regions). It must be borne in mind that the two micronutrients Zn and Ni are involved in different physiological processes [9,35]. Zinc is required for the activity of a series of enzymes (e.g., carbonic anhydrase, alcohol dehydrogenase, metalloproteinase, Cu-Zn superoxide dismutase), while nickel is essential for urease activity (an enzyme important for purine catabolism releasing ammonium from urea) [9,35,36,37].

Shoot growth was negatively influenced when the roots were subjected to drought stress even when parts of the root system were not stressed (Figure 1). This effect might be caused by the production of abscisic acid in stressed roots and its transport via the xylem to the shoot causing stomatal closure, although sufficient water may be delivered from control roots to the shoot [38]. Under such conditions, transpiration and CO_2_ assimilation may be lowered and, as a consequence, biomass production may be negatively influenced. 

The rapid transport of ^63^Ni from the shoot to the unlabeled roots is consistent with the previously reported good mobility of Ni in the phloem in unstressed plants [14,15,16,17]. In addition to previous knowledge, the transport from drought-stressed (+PEG) to control roots and from control to drought-stressed roots was addressed in the experiments reported here. The transfer of Rb from stressed to unstressed roots was significantly higher than in the opposite direction (Figure 4). The same trends were observed for Ni and Zn, although the differences were not significant. These findings indicate that the unstressed roots represented stronger phloem sinks than the stressed roots.

## 3. Materials and Methods

### 3.1. Plant Material and Culture Conditions

*Solanum nigrum* L. seeds were germinated as reported previously [17]. Briefly, dry seeds were soaked for one day in 0.1% (w/v) KNO_3_ at 4 °C and then placed on tissue paper moistened with 0.1% (w/v) KNO_3_. After 7 d, the seedlings were transferred to standard nutrient medium [17]. Plants were exposed to a light/dark cycle with 14 h light (200 µE m^−2^ s^−1^; 25 °C) and 10 h darkness (20 °C). The root system was removed by a horizontal cut in the hypocotyl 20 (for autoradiography) or 21 (for γ-spectrometry) days after the beginning of germination. The part of the hypocotyl attached to the shoot was split with a vertical cut into two equal parts before the shoots were brought back to standard nutrient medium. This treatment led to the formation of a split root system with two equally developed parts. The labeling experiment for γ-spectrometry was started 38 d after seed soaking, while smaller plants (28 days after seed soaking) were labeled for autoradiography.

### 3.2. Experimental Setup and Quantification of Labels

Two rectangular plastic containers with 150 mL standard nutrient medium were used for each set containing two plants with a split root system. When indicated (+PEG), 15 g polyethylene glycol 6000 were added to the 150 mL nutrient medium to lower the water potential [39,40]. The calculated initial water potential of the +PEG solution was −0.16 MPa [39,40]. The labels (Rb as RbCl, Sr as SrCl_2_, ^109^Cd, ^63^Ni, ^54^Mn and ^65^Zn) were added only to one of these containers as indicated. The plants used for this experiment were 38 d old. Rb and Sr were added at the beginning of the labeling phase (Day 0). The radionuclides were added from aqueous stock solutions 1 d (^109^Cd), 2 d (^63^Ni, ^54^Mn) or 3 d (^65^Zn) after the start of the labeling phase. The incubation conditions during the labeling phase were identical with those described above under Section 3.1. All plants were collected at Day 4 (4 d after the beginning of the labeling phase) and separated into shoot, labeled roots and unlabeled roots. The samples were dried in polystyrene tubes in an oven at 60 °C.

The γ-emitting radiolabels ^109^Cd, ^54^Mn and ^65^Zn were quantified in the dried plant samples simultaneously with a γ-spectrometer (1480 Wizard 3’, Wallac Oy, Turku, Finland). Afterwards, the dry weights of the samples were taken, and the plant material was transferred to glass tubes for dry ashing (2 times for 8 h at 550 °C), as described by Page and Feller [14]. After cooling, 0.3 mL 10 N HCl were added to each tube. After mixing, 3 mL deionized H_2_O were added. For the quantification of the β-emitter ^63^Ni, 0.2 mL of the well-mixed solutions were transferred to Ready Caps (Beckman Instruments, Fullerton, CA, USA). After drying the samples for 4 h at 60 °C, they were analyzed in a scintillation counter. The same solutions as for ^63^Ni were used for the detection of Rb and Sr by atomic spectrometry after appropriate dilution with 1000 ppm Cs as CsCl in 0.1 N HCl (for Rb) or with 5000 ppm La as LaCl_3_ in 0.1 N HCl (for Sr) as reported previously [11,14].

Means and standard errors of 6 replicate plants are shown in the figures. Significant differences between different treatments were identified with Student’s t-test. Columns with the same letter (a–c) are not significantly different at the *p* < 0.05 level.

### 3.3. Autoradiography and Localization of ^63^Ni and ^65^Zn

The labeling phase for autoradiography started when the plants were 28 d old and separate plants were used for the two radionuclides ^63^Ni and ^65^Zn. The same experimental setup as mentioned above under Section 3.2 was used, but the radionuclides were present in the nutrient medium for one part of the root system from the beginning of the labeling period of 4 d. Rb and Sr were not added to the nutrient medium. After the labeling phase, the plants were dried with tissue paper keeping the various parts of the root system well separated and then completely dried between several layers of tissue paper by ironing. The dry plants were the mounted on cardboard for the exposure to an x-ray film for 5 months in darkness.

## 4. Conclusions

Artificial drought strongly decreased the uptake of most elements considered in the plant and the release to the shoot, while the transfer to unlabeled roots was very low in *Solanum nigrum* and far less influenced by the water potential. Ni was an exception, since it was well redistributed and transported via the phloem to the unlabeled roots. No drastic effects of the water potential in the medium on the sink strength of unlabeled roots were detected after an incubation period of four days. However, there was a significant difference (Rb) and a clear trend (^63^Ni and ^65^Zn) in favor of the supply of unstressed roots. This difference might become more relevant during longer exposure times and might be involved in better root growth in soil regions with a less negative water potential.

Autoradiographs allowed the visualization of the radionuclide distribution in the roots and in the shoot. The different distribution patterns for ^63^Ni and ^65^Zn remain to be investigated in more detail in future experiments, since it was observed in various plant species and was highly reproducible in *Solanum nigrum* as well as in wheat or legumes [14,16]. The anatomy of roots and shoots may explain the observed differences [41]. ^63^Ni and ^65^Zn are easily transported in the phloem [14,15,16,17]. Therefore, the long-distance transport is presumably very similar, but in the root tip, in the shoot apex and in axillary buds, the phloem is not yet differentiated and can therefore not yet be functional. In these regions, the ions must be transported from cell to cell, and it must be hypothesized that this short-distance cell-to-cell transport is different for ^63^Ni and ^65^Zn. The mechanisms involved in Ni and Zn transport at the cellular and subcellular level are not yet clear and their elucidation represents a challenge for further investigations.
